# Supplementary material for: Prognostic Implications of Portal Venous Circulating Tumor Cells in Resectable Pancreatic Cancer
Source: Biomedicines. 2022 May 31;10(6):1289. doi: 10.3390/biomedicines10061289 (PMC9219704; doi:10.3390/biomedicines10061289)
Supplement: Supplementary file 1 [file biomedicines-10-01289-s001.zip › Supplemenatary Table S1.pdf]

Supplementary Table S1. Characteristics of individual patients and number of CTCs.

No.	Age	Sex	CA 19-9	Surgery	Primary tumor size (cm)	No. of LN metastases	Tumor differentiation	Final Stage	Adjuvant CTX	Palliative CTX.	Peripheral CTCs	Peripheral E-CTCs	Peripheral M-CTCs	Portal CTCs	Portal E-CTCs	Portal M-CTCs
1	43	F	25	PD	4.7	2	Moderate	IIB	Yes	Yes	1	0	1	6	4	2
2	50	M	383	DP	4.9	3	Moderate	IIB	No	Yes	2	0	2	15	1	14
3	71	F	18	PD	3.5	2	Moderate	IIB	Yes	Yes	4	0	4	10	3	7
4	81	F	2639	No	4.2	NA	Moderate	IV	No	Yes	9	2	7	NA	NA	NA
5	63	M	25	PD	3.2	0	Moderate	IB	Yes	Yes	2	1	1	1	0	1
6	40	M	135	PD	1.5	0	Well	IA	Yes	Yes	0	0	0	2	0	2
7	76	F	16391	No	3.2		Moderate	IV	No	No	3	0	3	NA	NA	NA
8	60	M	81	PD	3.7	0	Moderate	IB	Yes	Yes	8	6	2	8	2	6
9	56	M	135	No	3.5	NA	Well	III	No	Yes	5	3	2	NA	NA	NA
10	67	F	326	PD	2.5	3	Well	IIB	Yes	Yes	5	5	0	18	17	1
11	66	F	392	DP	7.0	5	Well	III	Yes	Yes	2	0	2	2	1	1
12	58	F	1221	No	4.5	NA	Moderate	IV	No	Yes	2	2	0	NA	NA	NA
13	49	M	858	PD	4.0	1	Moderate	IIB	Yes	Yes	1	0	1	7	0	7
14	70	F	83	PD	1.7	0	Well	IB	Yes	Yes	1	1	0	2	0	2
15	81	M	64	PD	6.3	0	Well	IA	Yes	Yes	0	0	0	0	0	0
16	75	F	891	PD	2.9	9	Well	III	Yes	Yes	4	4	0	15	15	0
17	65	F	752	PD	4.3	2	Moderate	IIB	Yes	Yes	1	1	0	1	0	1
18	48	F	30	PD	4.8	0	Moderate	IIA	Yes	Yes	3	0	3	0	0	0
19	46	M	10	PD	4.7	1	Poor	IIB	Yes	Yes	0	0	0	0	0	0
20	58	M	11	No	3.0	NA	Moderate	IV	No	No	2	0	2	NA	NA	NA
21	69	M	1988	PD	3.4	9	Poor	III	Yes	Yes	0	0	0	0	0	0

22	71	F	1279	PD	3.0	4	Moderate	III	Yes	Yes	1	0	1	3	1	2
23	69	F	2	PD	2.4	0	Well	IB	Yes	Yes	1	1	0	0	0	0
24	73	F	9	PD	3.5	0	Moderate	IB	Yes	Yes	1	0	1	1	1	0
25	58	M	817	PD	4.8	0	Poor	IIA	No	Yes	0	0	0	5	5	0
26	43	F	53	DP	3.5	4	Poor	IIA	Yes	Yes	2	0	2	7	6	1
27	60	F	463	PD	5.4	2	Poor	IIB	No	No	0	0	0	5	0	3
28	62	F	450	PD	2.4	0	Well	IB	Yes	Yes	2	0	2	1	0	1
29	61	M	1066	PD	4.3	7	Poor	III	Yes	Yes	2	0	2	18	16	2
30	75	M	22	PD	3.5	2	Moderate	IIB	Yes	Yes	0	0	0	1	1	0
31	65	F	590	PD	2.4	0	Well	IB	Yes	Yes	0	0	0	2	0	0
32	69	M	141	DP	1.8	1	Moderate	IIB	Yes	Yes	5	0	5	10	0	10
33	84	F	64	PD	2.5	2	Moderate	IIB	No	No	1	0	1	3	3	0

E-CTCs, epithelial circulating tumor cells; F, female; CTCs, circulating tumor cells; DP, distal pancreatectomy, LN, lymph node; M, male; M-CTCs, mesenchymal circulating tumor cells; Moderate, moderately differentiated, NA, Not available; PD, pancreaticoduodenectomy; poor, poorly differentiated, Well, well-differentiated.
