# Supplementary material for: Prognostic Implications of Portal Venous Circulating Tumor Cells in Resectable Pancreatic Cancer
Source: Biomedicines. 2022 May 31;10(6):1289. doi: 10.3390/biomedicines10061289 (PMC9219704; doi:10.3390/biomedicines10061289)
Supplement: Supplementary file 1 [file biomedicines-10-01289-s001.zip › Supplemenatary Table S2.pdf]

Supplementary Table S2. Comparison of characteristics with epithelial-CTC dominant type (E-type) *vs.* mesenchymal CTC dominant type (M-type) patients from portal venous blood.

Parameters	Subtypes		
	E-type (n = 9)	M-type (n = 13)	p value
**Patient Characteristics**			
Age, mean ± SD, years	64.3 ± 14.4	62.6 ± 10.2	0.572
Sex, male (%)	3 (33.3%)	6 (41.2%)	0.548
CA 19-9, median (IQR), (U/mL)	64 (24–854)	383 (82–671)	0.891
**Tumor Characteristics**			
Stage			0.193
Stage I	1 (11.1%)	6 (46.2%)	
Stage II	6 (66.7%)	6 (46.2%)	
Stage III	2 (22.2%)	4 (7.7%)	
Primary tumor size, mean ± SD, cm	3.6 ± 0.8	3.2 ± 1.2	0.458
Regional LN involvement (%)	7 (77.8%)	7 (53.8%)	0.251
Tumor differentiation			0.309
Well-differentiated (%)	2 (22.2%)	4 (30.8%)	
Moderately differentiated (%)	4 (44.4%)	8 (61.5%)	
Poorly differentiated (%)	3 (33.3%)	1 (7.7%)	
