# Supplementary material for: Prognostic Implications of Portal Venous Circulating Tumor Cells in Resectable Pancreatic Cancer
Source: Biomedicines. 2022 May 31;10(6):1289. doi: 10.3390/biomedicines10061289 (PMC9219704; doi:10.3390/biomedicines10061289)
Supplement: Supplementary file 1 [file biomedicines-10-01289-s001.zip › Supplementary Figure S1.pdf]

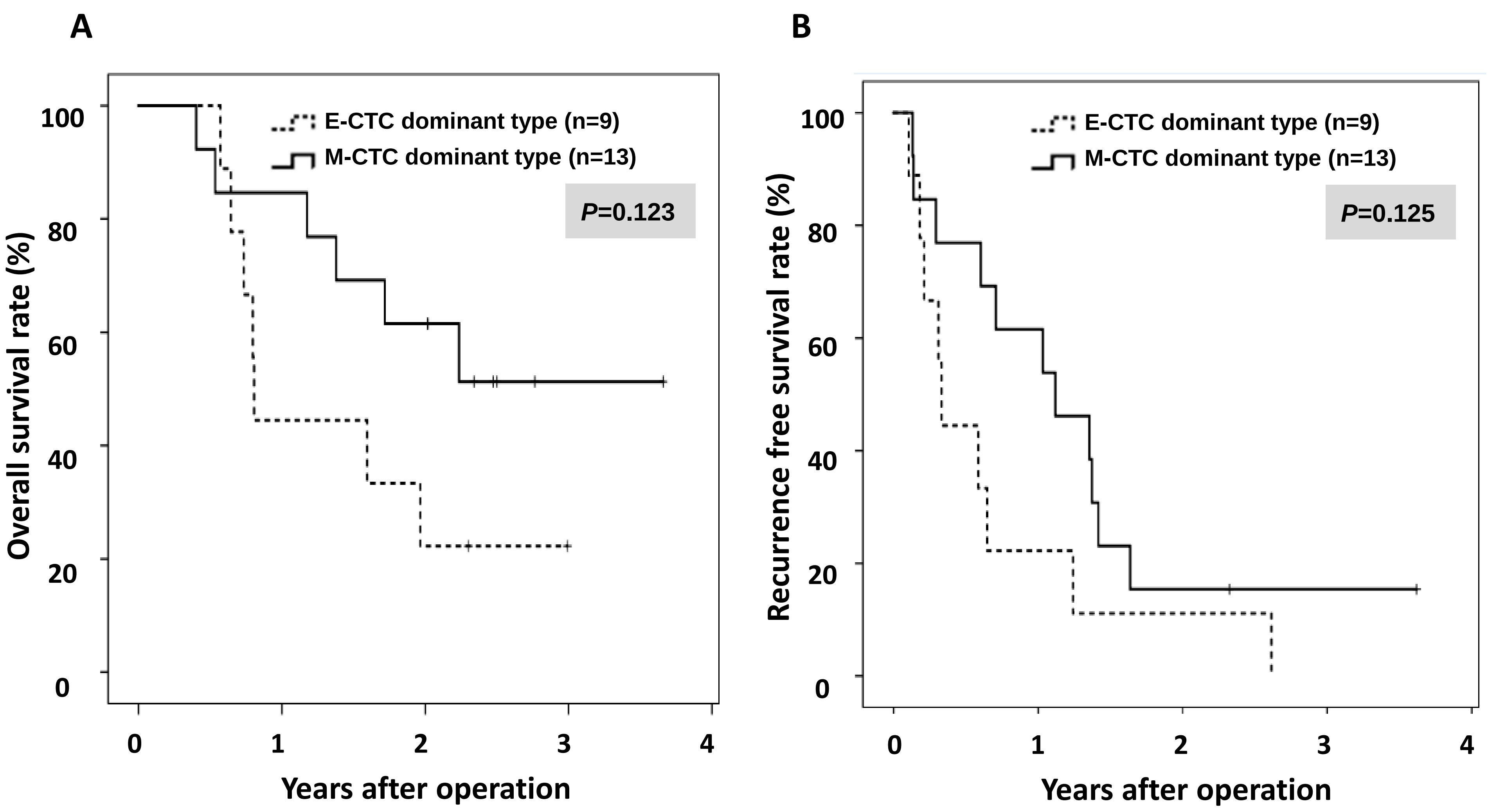

Figure S1. Comparison of survival between epithelial-CTC dominant type (E-type) and mesenchymal CTC dominant type (M-type) patients from portal venous blood. Phenotype of CTCs was not associated with overall survival (A) and recurrence free survival (B) after resection of pancreatic cancer
